# Global detection and management of dysglycaemic patients with coronary artery disease results from the INTERASPIRE survey from 14 countries across six WHO regions

**DOI:** 10.1186/s12933-025-02878-3

**Published:** 2025-08-11

**Authors:** Safi Moayad Al-Azzawy, John William McEvoy, Isabelle Johansson, Agnieszka Adamska, Guy De Backer, Iris Erlund, Sandra Ganly, Catriona Jennings, Kornelia Kotseva, Gregory Y. H. Lip, Linda Mellbin, Kausik K. Ray, Terhi Vihervaara, David Wood, Ana Abreu, Wael Almahmeed, Ade Meidian Ambari, Junbo Ge, Hosam Hasan-Ali, Yong Huo, Piotr Jankowski, Rodney M. Jimenez, Yong Li, Syadi Mahmood Zuhdi, Abel Makubi, Amam Chinyere Mbakwem, Lilian Mbau, Jose Luis Navarro Estrada, Okechukwu Samuel Ogah, Elijah Nyainda Ogola, Adalberto Quintero–Baiz, Mahmoud Umar Sani, Maria Ines Sosa Liprandi, Jack Wei Chieh Tan, Miguel Alberto Urina Triana, Tee Joo Yeo, Dirk De Bacquer, Lars Rydén

**Affiliations:** 1 Department of Medicine Solna, Karolinska Institutet, FoU–Tema Hjärta och Kärl, S1:02, Karolinska Universitetssjukhuset/Solna, SE-171 76c Stockholm, Sweden; 2 University of Galway School of Medicine and National Institute for Prevention and Cardiovascular Health, Moyola Lane, Newcastle, Galway, H91 FF68 Ireland; 3 Department of Public Health and Primary Care, Ghent University, Corneel Heymanslaan 10, UZ Ghent—4K3, 9000 Gent, Belgium; 4 Institute for Nutrition and Health Research FI, Helsinki, Finland; 5 Liverpool Centre for Cardiovascular Science at University of Liverpool, Liverpool John Moores University and Liverpool Heart & Chest Hospital, Liverpool, UK; 6 Danish Center for Health Services Research, Department of Clinical Medicine, Aalborg University, Aalborg, Denmark; 7 Department of Public Health and Primary Care, Imperial College London, London, UK; 8 Cardiology Service, Cardiovascular Rehabilitation Unit, Hospital S. Maria (ULSSM), Faculty of Medicine, University of Lisbon, CAML, ISAMB, IMPSP, CCUL, Lisbon, Portugal; 9 Cleveland Clinic Abu Dhabi, Al Mariah Island, Abu Dhabi, United Arab Emirates; 10 Heart and Vascular Institute, Cleveland Clinic Abu Dhabi, Al Mariah Island, Abu Dhabi, United Arab Emirates; 11 National Cardiovascular Center Harapan Kita, Department of Cardiology and Vascular Medicine, Faculty of Medicine, Universitas Indonesia, West Jakarta, Jakarta,, Indonesia; 12 Fudan University Zhongshan Hospital, Floor 15, Building 16, No. 1609 Xietu Road, Xuhui District, Shanghai, 200000 China; 13 Cardiovascular Medicine Department, Assiut University Heart Hospital, Faculty of Medicine, Assiut University, Assiut, 71111 Egypt; 14 Department of Cardiology, Institute of Cardiovascular Disease, Peking University First Hospital, No. 8 XiShiku Street, Xicheng District, Beijing, 100034 China; 15 Department of Internal Medicine and Geriatric Cardiology, Centre of Postgraduate Medical Education, 231 Czerniakowska St, 00-416 Warsaw, Poland; 16 Philippine Heart Association and St. Luke’s Medical Center, Global City, Rizal Avenue Corner 32nd Street, Taguig City, Metro Manila, Philippines; 17 Department of Internal Medicine (Cardiology), Fudan University Huashan Hospital, 12 Wulumuqi Zhong Rd, Shanghai, People’s Republic of China; 18 Cardiology Unit, Department of Medicine, University Malaya Medical Centre, 59100 Kuala Lumpur, Malaysia; 19 Muhimbili University of Health and Allied Sciences (MMUHAS), P.O BOX 65001, Dar Es Salaam, Tanzania; 20 College of Medicine, University of Lagos, Idi Araba, Lagos, Nigeria; 21 Kenya Cardiac Society, P.O Box 8038–00100, Nairobi, Kenya; 22 Italian Hospital of Buenos Aires, Gascon 450, 1181 Buenos Aires, Argentina; 23 Cardiology Unit, Department of Medicine, University of Ibadan, University College Hospital, Ibadan, Nigeria; 24 Dep, University of Nairobi, P. O. Box 19676, Nairobi, Kenya; 25 Faculty of Health Sciences, University Simon Bolivar, Cra 50 # 80–216 office 109, Barranquilla, Colombia; 26 Department of Medicine, Bayero University Kano & Aminu Kano Teaching Hospital, Kano, 700001 Kano State Nigeria; 27 Cardiology Service, University Hospital Sanatorio Güemes, Francisco Acuna de Figueroa, 1240, 1180 Buenos Aires, Argentina; 28 Duke-NUS Medical School, National Heart Centre Singapore, Singapore, Singapore; 29 Faculty of Health Sciences, Universidad Simon Bolivar, Carrera 59 # 59-65, Barranquilla, Colombia; 30 Cardiac Department, National University Heart Centre Singapore and National University Hospital, National University Health System (NUHS) Tower Block, 1E Lower Kent Ridge Road, Level 9, Singapore, 119228 Singapore

**Keywords:** Screening, Dysglycaemia, Type 2 diabetes, Coronary artery disease, Cardiovascular prevention, Diabetes education program

## Abstract

**Objective:**

Dysglycaemia, defined as type 2 diabetes mellitus (T2DM) or impaired glucose tolerance (IGT), increases the cardiovascular risk and prognosis. INTERASPIRE performed in 14 countries across 6 WHO regions evaluated guideline adherence and management of patients with coronary artery disease (CAD) and dysglycaemia.

**Methods:**

A total of 4,548 CAD patients (18–80 years) were interviewed 6 months–2 years after hospital admission. All without diabetes were eligible for an oral glucose test (OGTT).

**Results:**

Overall, 1990 (44%) had known T2DM. The OGTT revealed that 808 (40%) had previously unknown dysglycaemia (T2DM 12% and IGT 28%). Two thirds of all dysglycaemic patients were obese. A similar proportion reported low physical activity and only one third received dietary advice. Only half of dysglycemic patients were prescribed all guideline recommended cardioprotective drugs. A majority did not reach recommended blood pressure, lipids or HbA1c targets. Only 16% had attended a diabetes education program.

**Conclusions:**

The INTERASPIRE study shows that screening for glucose perturbations in coronary patients is inadequate, achievement of lifestyle recommendations suboptimal and pharmacological management insufficient resulting in a poor risk factor control. Patients with coronary disease, especially those with glucose perturbations require professional support to achieve healthier lifestyles, and prescription of all cardioprotective medications to achieve guideline targets.

**Supplementary Information:**

The online version contains supplementary material available at 10.1186/s12933-025-02878-3.

## Research insights

**What is currently known about this topic**

Patients with coronary artery disease do often have undetected dysglycaemia (type 2 diabetes or impaired glucose tolerance). The presence have dismal prognostic implications.

**What is the key research question**

To determine if international guidelines for screening and management of dysglycaemia in coronary patients are adhered to in a global perspective.

**What is new**

The study shows that screening for dysglycaemia is inadequate, achievement of guideline-recommended management is insufficient, resulting in an increased risk for future cardiovascular events.

**How might this study influence clinical practice?**

Increase the awareness that patients with coronary disease should be screened for glucose perturbations and receive professional support to achieve healthier lifestyles, and prescription of cardioprotective medications to achieve guideline targets.

## Introduction

In low- and middle-income countries patients below the age of 70 years are at a higher risk for death due to cardiovascular disease (CVD), often due to coronary artery disease (CAD) [1]. Indeed, CVD remains a leading cause of global mortality and disability, causing 20 million deaths in 2021, whereof about 80% occurred in low- and middle-income countries. About 85% of these deaths are related to coronary artery disease or stroke [1]. Global trends indicate an absolute increase in the number of deaths, mostly due to a growing population and an increased ageing while the age-standardized incidence has decreased. This decline is most apparent in high-income countries while it is less or not at all apparent in low- and middle-income countries with almost no improvement in death rates for men in these regions [2].

Dysglycaemia, defined as type 2 diabetes mellitus (T2DM) or impaired glucose tolerance (IGT), is among the most important risk factors for de novo or recurrent CVD [3–6]. Accordingly, international guidelines uniformly underline the necessity to screen patients with CAD for glucose perturbations to ensure that those with dysglycaemia are offered appropriate, multifactorial management including lifestyle oriented and pharmacologic interventions all essential for their prognosis [7, 8].

Reports from the European Action on Secondary and Primary Prevention by Intervention to Reduce Events (EUROASPIRE) cross-sectional surveys show that both screening and management of patients with CAD and dysglycaemia is far from perfect in Europe [9–11]. Similar findings have been reported in the US [12]. Analogous reports regarding low- and middle-income countries are limited, but according to a recent investigation by Flood et al. [13] less than 10% of persons with T2DM in such countries received a comprehensive, guideline-based management in 2021 [13].

INTERASPIRE, an international study of secondary prevention of patients with CAD, was performed in collaboration with the World Heart Federation and other scientific societies in 14 member countries from the six WHO regions: Africa (Kenya, Nigeria and Tanzania), Americas (Argentina and Colombia), Eastern Mediterranean Region (Egypt and UAE), Europe (Poland and Portugal), South-East Asia (Indonesia) and Western Pacific Region (China, Malaysia, Philippines, Singapore). The study was conducted through national societies of cardiology based on the principles of the EUROASPIRE programme [14].

The objective of this report is to determine if international guidelines for screening and management of dysglycaemic patients are adhered to.

## Material and methods

A detailed description of the study protocol for INTERASPIRE has been presented elsewhere [15]. In brief, 4548 (21% women) consecutive patients, 18–80 years of age with a first or recurrent coronary event (elective or emergency CABG, elective or emergency PCI, acute myocardial infarction, and unstable angina/acute myocardial ischaemia) were identified. They were invited to a study visit at one of the 88 participating hospitals across 14 countries from the six WHO regions between six months and two years after the index event (except for Africa where the timeframe was extended to 36 months to enable recruitment).

### Data collection

Data were collected by centrally trained research staff using standardised methods and instruments. Information on their case history including diabetes related complications (retino-, nephro- and neuropathy), demographics, health behaviours, CVD risk factor management and all prescribed medication was obtained from their medical records and from the patient interviews. The following standardised assessments were performed at the time of the study visit:

*Smoking* was defined as self-reported smoking, and/or a breath carbon monoxide exceeding 10 ppm by means of Smokerlyzer (Bedfont Scientific, Model Micro +). Persistent smoking was defined as smoking at the time of the interview among those who smoked in the month prior to the index event.

*Height and weight* were measured in light indoor clothes without shoes using a calibrated measuring stick and scale. Overweight was defined as a body mass index (BMI) ≥ 25 to < 30 kg/m^2^ and central distribution of adiposity as a BMI ≥ 30 kg/m^2^.

*Waist circumference* was measured with the patient standing using a metal tape applied at the point between the lowest rim of the rib cage and the tip of the superior iliac crest. Central obesity was defined as a waist circumference of ≥ 88 cm for women and ≥ 102 cm for men.

*Blood pressure* was measured twice on the right upper arm after five minutes in a sitting position by means of a digital sphygmomanometer (Omron Health Care, Kyoto Japan) using the mean of both measurements for the analyses.

*Venous (fasting) blood* was drawn in a sitting position for serum total (TC) and high-density lipoprotein cholesterol (HDL-C), triglycerides (TG), and creatinine. Fasting glucose was measured in plasma, and glycated haemoglobin (HbA1c) in whole blood. Samples were taken with light stasis into a tube containing clot activator (Vacutainer SST II Advanced, Becton Dickinson) for lipid assays and creatinine, fluoride citrate sampling tube for plasma glucose (fasting and 2-h glucose) and a potassium EDTA tube (Vacutainer K2EDTA) for the HbA1c assay. After centrifugation serum and plasma were aliquoted and stored together with EDTA tubes with whole blood at a minimum of -70 °C. Low-density lipoprotein cholesterol (LDL-C) was calculated using Friedewald’s formula if the TG level was < 4.5 mmol/L (< 400 mg/dL). An oral glucose tolerance test (OGTT; 75 g glucose dissolved in 200 ml water) was performed in patients free from known diabetes with fasting and 2-h plasma glucose measured in the local laboratories.

*Level of care* was defined as care provider and proportion of patients reporting attendance at a diabetes education program.

The laboratory analyses for all countries apart from four were conducted at a Central Laboratory (Biomarkers Team, Department of Government Services, Finnish Institute for Health and Welfare [THL]), which is accredited by the Finnish Accreditation Service and fulfils the requirements of the standard SFS-EN ISO/IEC 17025:2005. Frozen blood samples were transported in dry ice from national laboratories to the Central Laboratory. All measurements were performed on a clinical chemistry analyzer (Architect c8000; Abbott Laboratories, Abbott Park, Illinois, US). The following methods were used: enzymatic methods for TC, HDL-C, creatinine, TG and HbA1c, and an enzymatic hexokinase method for glucose. The estimated glomerular filtration rate (eGFR) was estimated according to the Chronic Kidney Disease Epidemiology Collaboration equation (CKD-EPI) using the race-free definition [16].

The laboratory analyses of the samples from China, Egypt, Indonesia, and UAE were performed by an accredited laboratory in each country. Standardization of results between these national laboratories and the Central Laboratory in Helsinki was performed. Satisfactory laboratory standardization for the Egyptian samples could not be achieved, and therefore all data based on laboratory analyses from this country were excluded.

### Definitions

Previously known diabetes was defined as a self-reported history of T2DM or use of any glucose-lowering medication. Newly detected dysglycaemia (IGT or T2DM) based on an OGTT was defined according to the World Health Organization (Supplementary Table 1) [17]. No dysglycemia was defined as the absence of IGT or T2DM according to the OGTT. Elevated HbA1c in patients with diabetes was defined as ≥ 7.0% (≥ 53 mmol/mol). Impaired fasting glucose without elevated HbA1c was not considered as dysglycaemia.

### Data management

Data were collected electronically and submitted online to the data management center in the EURObservational Research Programme, European Heart House, Sophia Antipolis, France, for Argentina and Malaysia, and for the other countries, ARO Specialist Data Centre Services (previously AIMES), Liverpool, UK. Data were checked for completeness, internal consistency, and accuracy. All data were stored under the provisions of the national data protection regulations.

### Statistical analyses

The distributions of categorial and quantitative characteristics were summarized according to the frequencies, proportions, means and standard deviations (SD). The distributions of patient characteristics were compared between glycaemic categories by using the Mann–Whitney U-test for continuous variables and Fisher’s exact test for categorical variables. Statistical analyses were performed using SAS statistical software release V.9.4 (SAS Institute, Cary, NC) at the Department of Public Health and Prima Care, Ghent University.

## Results

### Glycaemic category

**Fig. 1:**
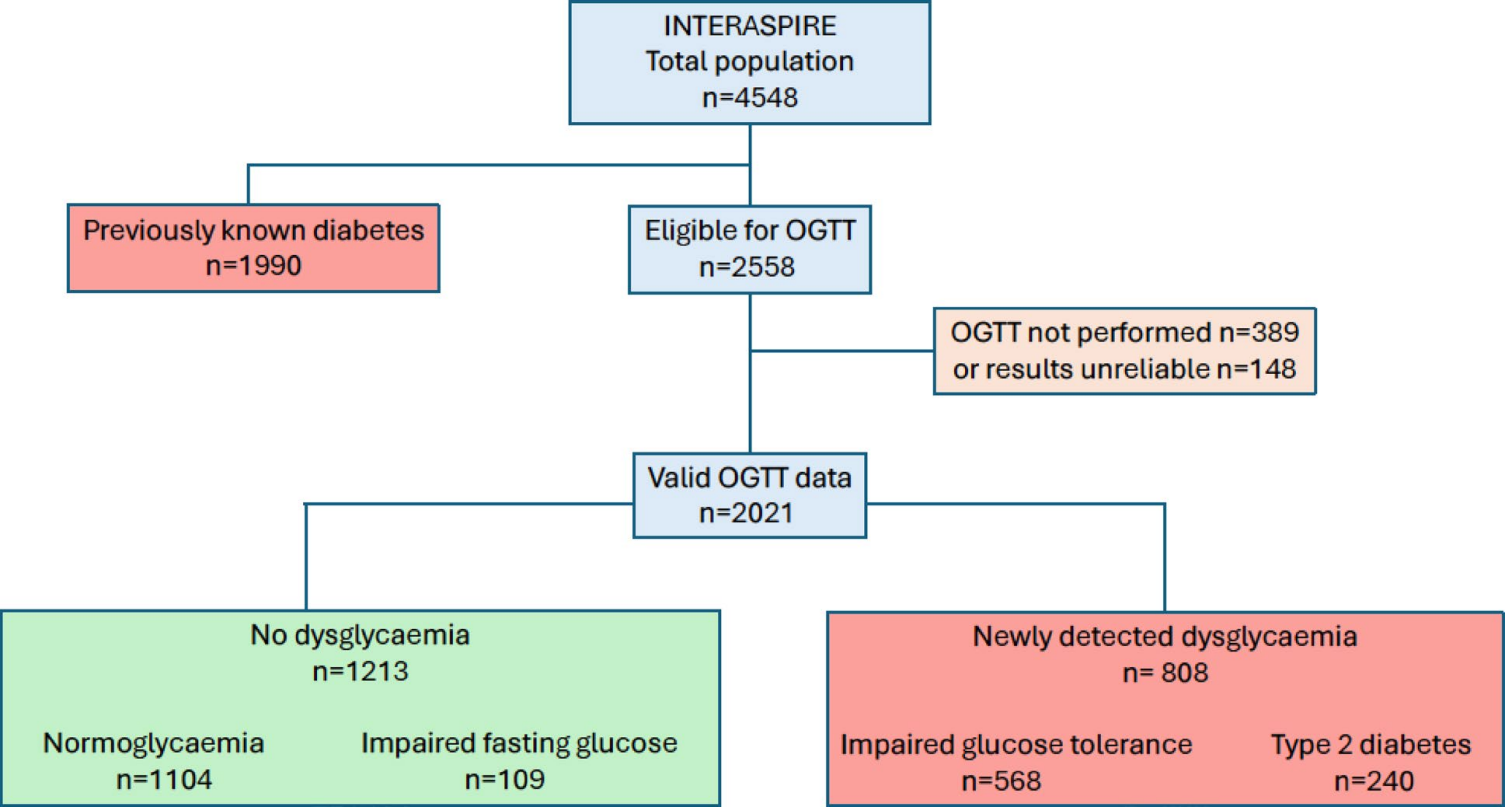
Patient flow by glycaemic state in INTERASPIRE

**Fig. 2:**
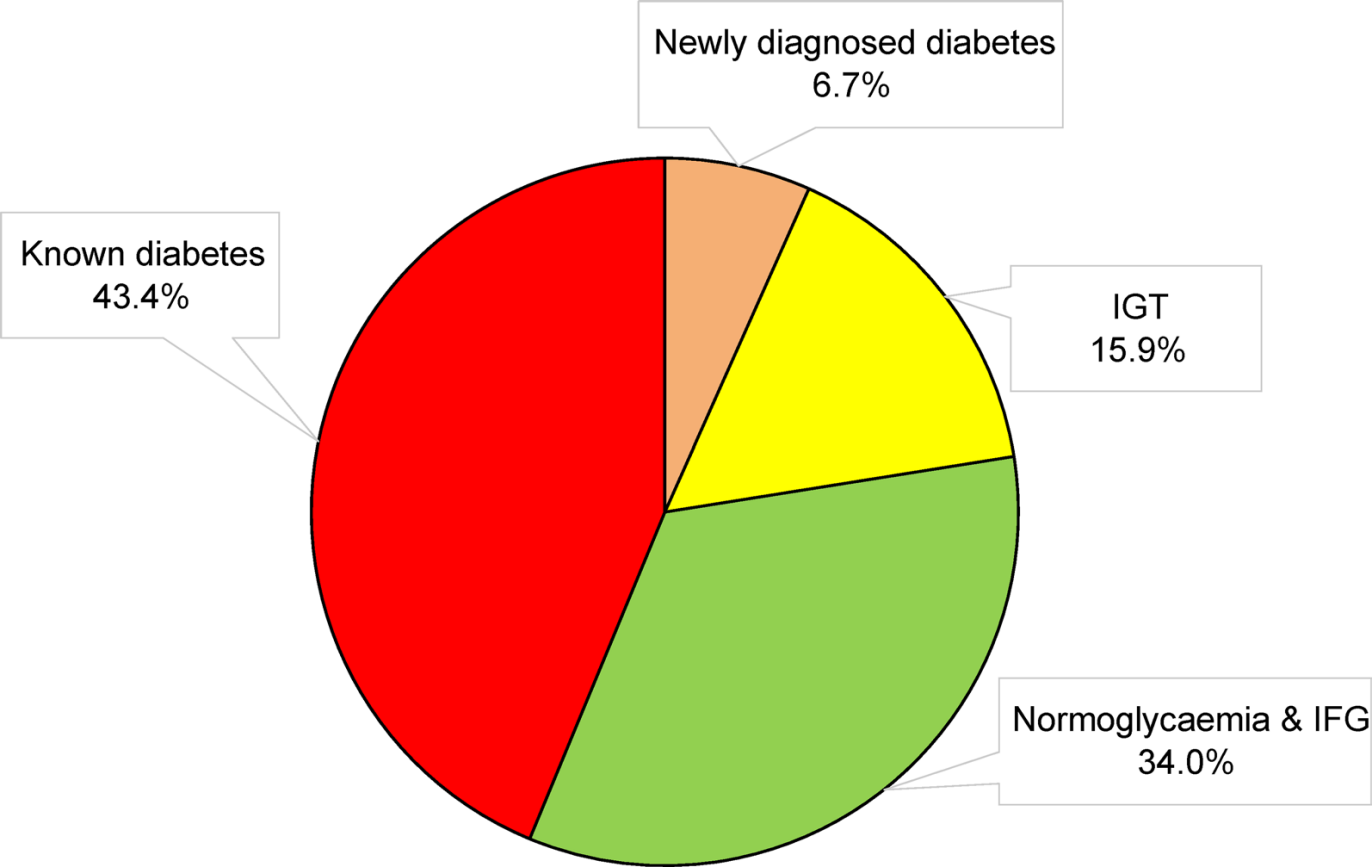
Distribution of glycaemic state in 3863 patients with coronary artery disease as revealed by case history and an Oral Glucose Tolerance Test performed on those without previously known dysglycaemia (all Egyptian patients excluded). *IGT* Impaired Glucose tolerance, *IFG* Impaired Fasting Glucose

Of the 4548 participants in INTERASPIRE 1990 (44%) had a diagnosis of diabetes or were prescribed glucose-lowering drugs at the time of interview leaving 2558 eligible for an OGTT. This test was either not performed (n = 389) or the outcome unreliable (n = 148 patients from Egypt) in 537 patients as outlined in Fig. 1. Of the 2021 patients with valid OGTT information 808 (40%) had newly detected dysglycaemia. The distribution of different glycaemic states based on the population of 3863 patients with known diabetes or OGTT results is presented in Fig. 2.

**Fig. 3:**
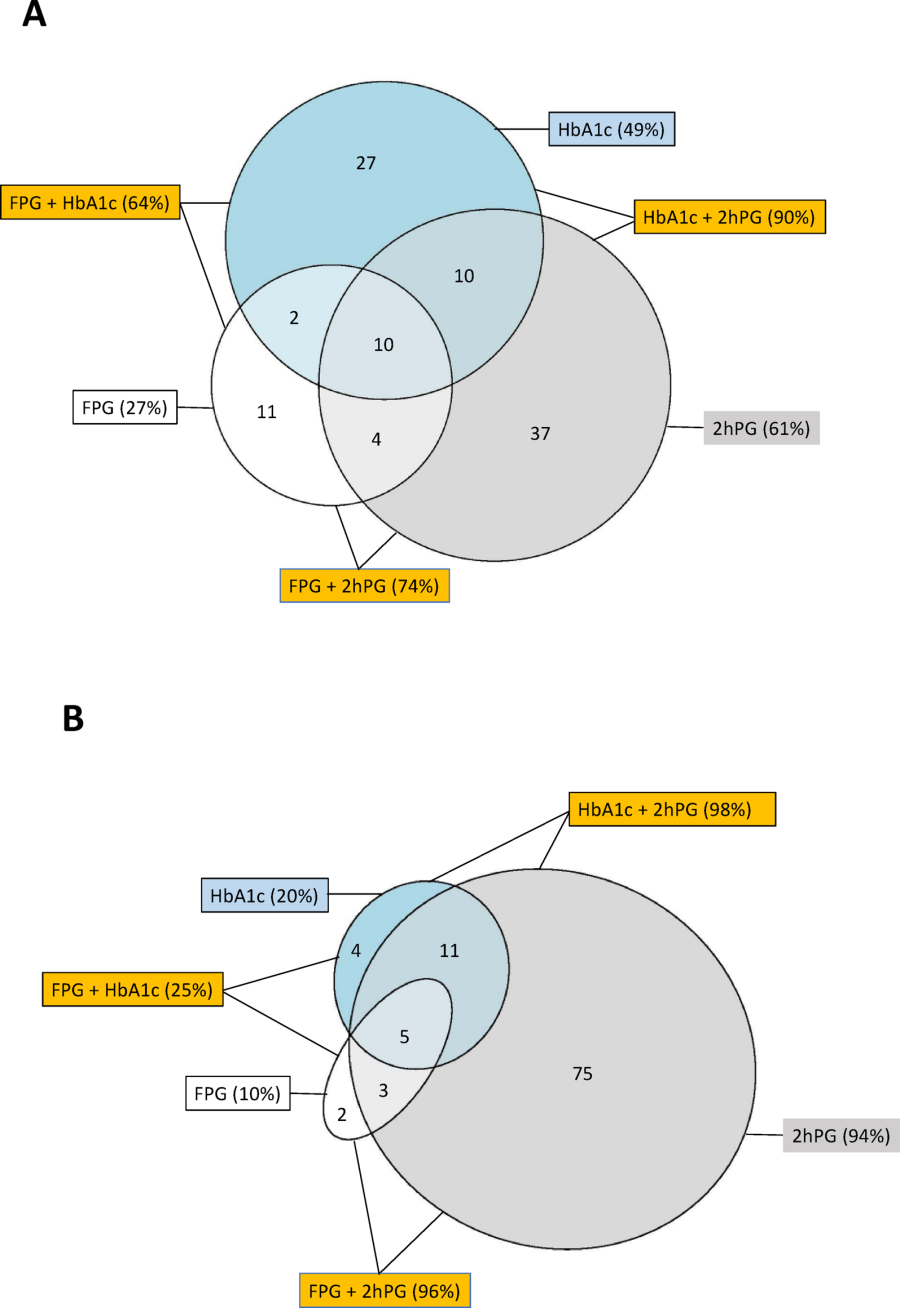
**A**: Proportions and their overlap between screening with FPG, 2hPG and HbA1c and their combinations in the 327 patients with newly detected type 2 diabetes. **B**: Proportions and their overlap between screening with FPG, 2hPG and HbA1c and combinations in the 808 patients with newly detected dysglycemia. *FPG* Fasting Plasma Glucose, *2hPG* Two-hour postload plasma glucose, *HbA1c* Glycated Hemoglobin A1c

Considering Fasting Plasma Glucose [FPG] ≥ 7.0 mmol/L and/or two-hour postload glucose [2hPG]) ≥ 11.1 mmol/L as identified by the OGTT with an elevated HbA1c ≥ 6.5% the total number of patients with newly detected T2DM was 327. The concordance of the three diagnostic tests is shown in Fig. 3A. Only 27% would have been detected if FPG had been the only test. The corresponding proportions were 61% for 2hPG and 49% for HbA1c. The highest yield, 90%, was obtained by means of a combination of 2hPG and HbA1c, with the remaining patients detected by FPG. The proportion identified as having T2DM by all tests was 10%. If an OGTT had been the only test, 27% of the patients with newly detected T2DM would have been missed.

Figure 3B shows the pattern for detection of the 808 patients with dysglycaemia (IGT or T2DM). The OGTT (FPG + 2hPG) detected 96% with HbA1c adding 4%. If HbA1c had been the only test 80% of the patients would have been missed

### Risk factor management, awareness and degree of care

Table 1 presents lifestyle characteristics including advice on weight loss, diet, physical activity, glycaemic variables and pharmacological treatment by the glycaemic state at the time for interview. About half of the 14% of the patients with known diabetes who were smokers at the time of their index event were persistent smokers i.e. still smokers at the time of the interview. Approximately two-thirds of the patients were overweight or obese which was not necessarily reflected on glycaemic state. Abdominal obesity was less common in normoglycaemic patients (30%) than in those with known or newly detected IGT or T2DM (43%, 38% and 38%). About 40% of those with obesity had not made any attempts to lose weight during the preceding month, and up to one-third of the patients had not received any dietary advice. Regardless of the glycaemic state approximately two-thirds of the patients reported a low physical activity, and up to 46% of them had not been instructed to increase activity.

**Table 1:** Clinical and life-style characteristics by glycaemic state according to the oral glucose tolerance test at the time for the interview. Numbers are % (n) or mean (SD) if not otherwise stated

Variables	Known T2DM^*^ n = 1849	Normal^**^ n = 1213	New IGT n = 568	New T2DM n = 240	Significance^***^
*Demographics*					
Age (years)	61.5 (9.7)	58.0 (10.6)	60.5 (10.8)	60.5 (10.0)	*P* < 0.0001
Gender (females)	22.7% (420)	18.2% (221)	23.4% (133)	22.5% (54)	*P* = 0.012
Low education	25.3% (466)	22.5% (271)	31.1% (176)	25.8% (62)	*P* = 0.0018
*Lifestyle characteristics*					
Current smoking	13.6% (252)	19.6% (238)	14.6% (83)	13.3% (32)	*P* = 0.0001
Persistent smoking	50.5% (230)	53.4% (219)	44.6% (79)	42.4% (28)	*P* = 0.14
Low physical activity (< 30 min, 5x/week)	64.0% (1184)	59.7% (724)	65.0% (369)	58.3% (140)	*P* = 0.027
Overweight or obesity	68.8% (1267)	65.5% (794)	69.9% (397)	59.2% (141)	*P* = 0.0067
Obesity	27.1% (500)	21.7% (263)	25.0% (142)	21.0% (50)	*P* = 0.0036
Abdominal obesity	42.7% (784)	29.8% (359)	37.7% (213)	38.0% (90)	*P* < 0.0001
Obese—no attempt lose weight last month	40.1% (194)	42.5% (110)	36.9% (52)	46.0% (23)	*P* = 0.018
Obese—not advised dietary guidelines	23.6% (116)	28.2% (73)	29.8% (42)	36.0% (18)	*P* = 0.12
Obese—not advised regular physical activity	26.4% (131)	34.2% (90)	34.3% (49)	46.0% (23)	*P* = 0.0053
*Glycaemic variables*					
FPG (mmol/L)	7.60 (3.29)	5.21 (0.63)	5.48 (0.66)	6.66 (1.67)	*P* < 0.0001
HbA1c (%)	7.31 (1.85)	5.65 (0.48)	5.93 (0.92)	6.33 (0.95)	*P* < 0.0001
*Pharmacological treatment*					
Antiplatelets/Anticoagulants	95.4% (1760)	94.3% (1143)	94.0% (531)	96.2% (230)	*P* = 0.31
Beta-blockers	79.5% (1466)	75.5% (913)	72.2% (407)	78.2% (187)	*P* = 0.0015
ACE- or Angiotensin receptor inhibitors	63.5% (1172)	63.7% (772)	66.7% (377)	67.8% (162)	*P* = 0.34
Lipid lowering drugs	90.6% (1671)	86.0% (1042)	84.1% (475)	90.0% (215)	*P* < 0.0001
All these four	48.1% (887)	45.7% (553)	45.0% (254)	52.3% (125)	*P* = 0.16

^*^Excluding patients with known diabetes from Egypt; **Including patients with impaired fasting glucose; ***According to Fisher’s exact test or Mann–Whitney U test

*T2DM* Type 2 Diabetes Mellitus, *IGT* Impaired Glucose Tolerance, *SD* Standard Deviation, *ACE* Angiotensin Converter Inhibitor

The proportion of patients prescribed cardioprotective drugs, antiplatelets, beta-blockers, ACE-inhibitors or ARBs and lipid lowering drugs was high in all glycemic groups. A combination of all four drug classes was, however, only reported by around half of the patients.

**Fig. 4:**
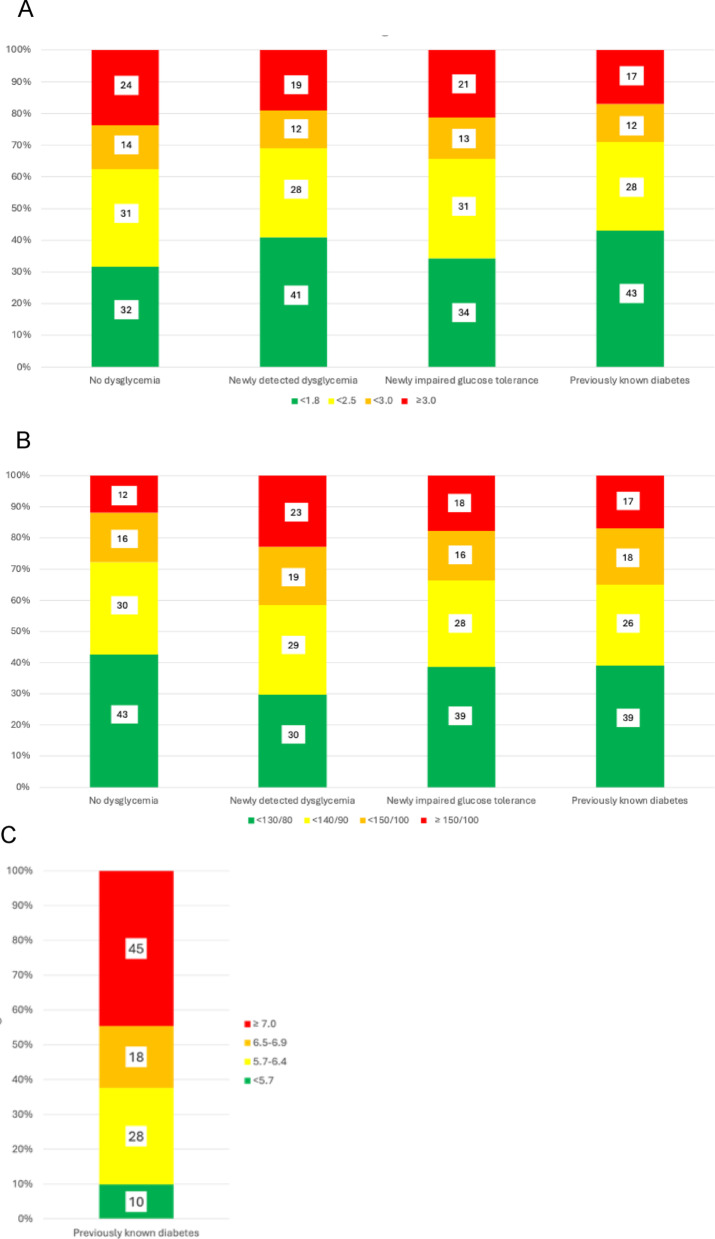
**A**: Proportion of patients reaching different LDL-C levels (Low Density Lipoprotein Cholesterol; mmol/l) by glucose category. **B**: Proportion of patients reaching different blood pressure levels (mmHg) by glucose category. **C**: HbA1c (Glycated Hemoglobin A1c; %) levels in patients with previously known diabetes

Figure 4 presents the proportion of patients who reached different laboratory risk factor targets by glycaemic state. Thirty-nine per-cent of those with known diabetes reached the recommended blood pressure target of < 130/80 mmHg. The corresponding proportions for those with newly discovered dysglycemia and for normoglycaemic patients were 36% and 43% respectively. The recommended LDL-C target < 1.8 mmol/L at the time of the study was reached in 32%, 34%, and 43% of normoglycaemic, newly detected dysglycaemic and T2DM patients respectively. Forty-five per-cent of the patients with known diabetes had a HbA1c level above the recommended 7.0% (53 mmol/mol).

### Glycaemic status by country and region

**Fig. 5:**
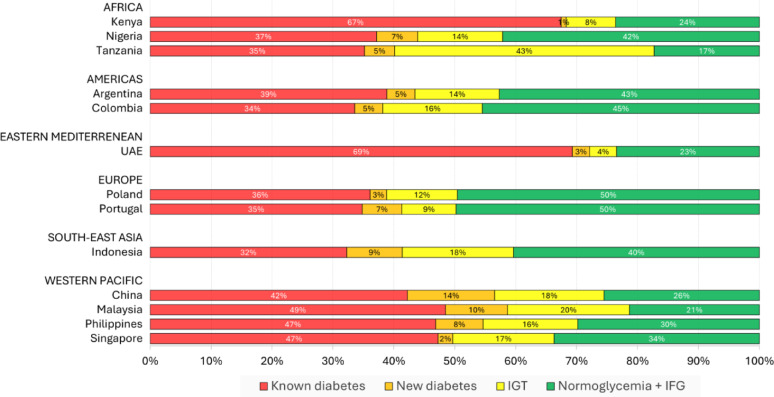
Glycemic state by region and countries.  = known diabetes; = new diabetes;  = impaired glucose tolerance;  = normoglycaemia and impaired fasting glucose

Glycaemic state by country and region is presented in Fig. 5. The proportion of self-reported T2DM, 32–39% in most countries, was considerably higher in Kenya (67%) and in the United Emirates (69%) and to some extent also in the countries from the Western Pacific area. Therefore, the proportion of newly detected dysglycaemia was lower in these countries/regions.

### Management and complications of diabetes

**Fig. 6:**
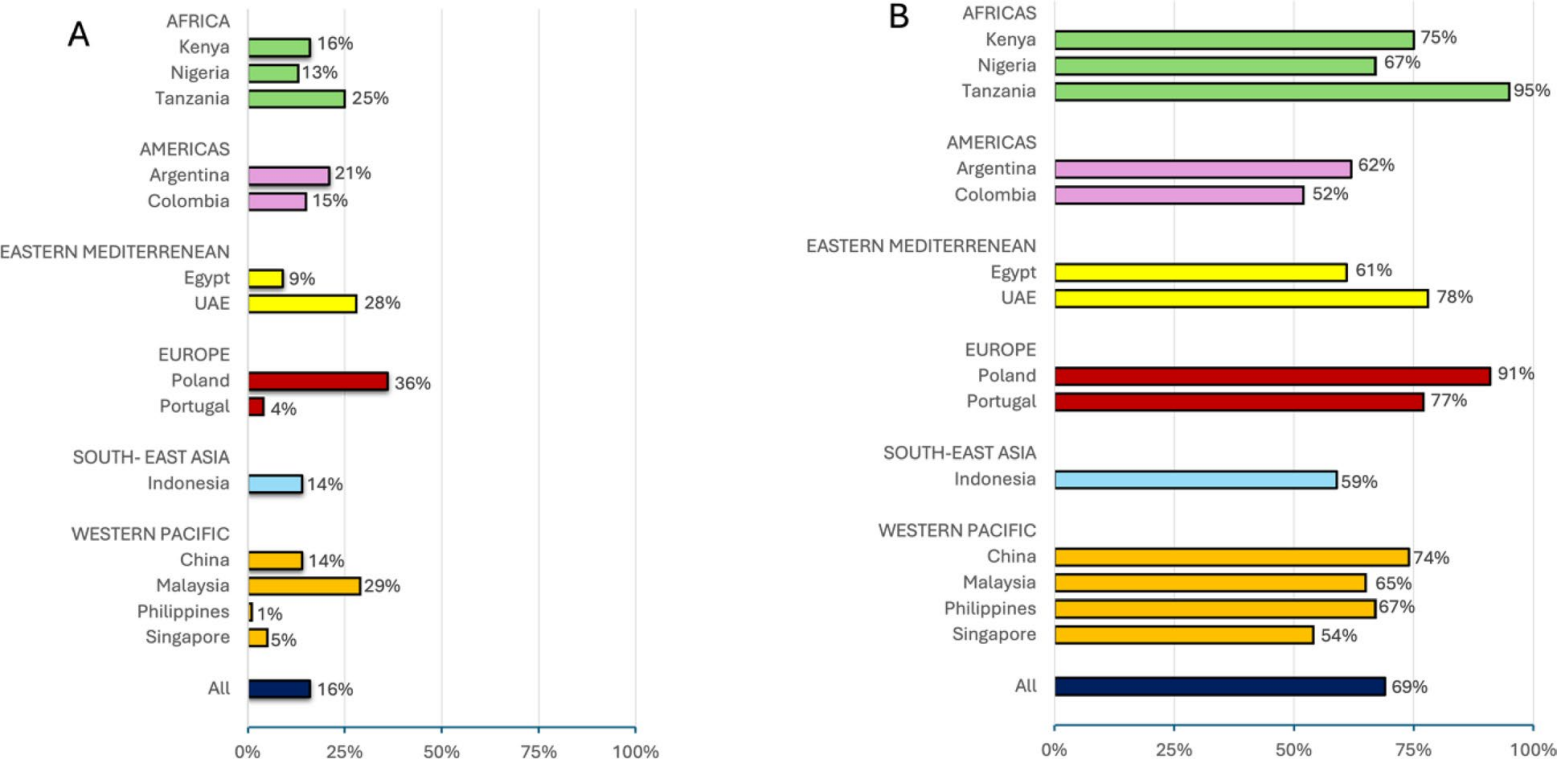
**A:** Proportion of patients with known diabetes attending a diabetes school, **B:** Proportion of patients with known diabetes who self-monitor their blood glucose

When asked during the interview 56% of the patients with known diabetes reported a positive family history of the disease. A majority, 88%, were cared for by a cardiologist, 23% by a primary care physician, 19% by an endocrinologist, 17% by an unspecified physician, 2% by a nurse and 4% did not report on any specific care provider. The proportion of patients with known diabetes reporting attendance at a diabetes education program, overall 16% and by country, is shown in Fig. 6A. The attendance ranged from 1 to 36% between countries while self-monitoring of blood glucose, 69%, in those with diabetes, varied between 52 and 95% (Fig. 6B). Self-monitoring was more common among women (325/426; 76%) than among men (925/1394; 66%; *p* < 0.0001, Fischer’s exact test).

**Fig. 7:**
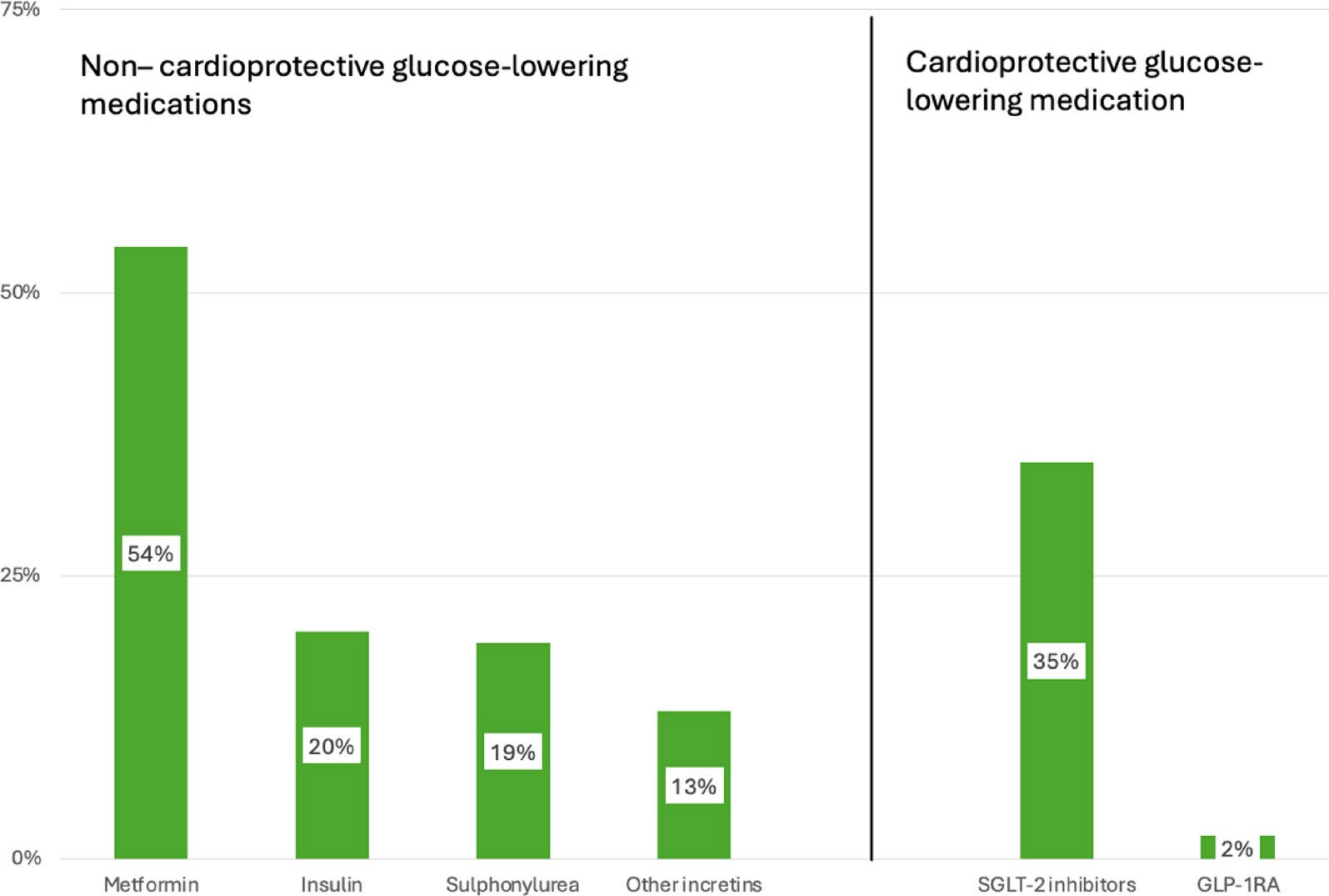
Prescription of different glucose lowering drugs to patients with diabetes. Data are shown as percent (%). Multiple prescriptions are possible explaining that columns add > 100%

The prescription of glucose lowering drugs in those with known diabetes is presented in Fig. 7. The most frequently used drug, metformin, was prescribed to 54% of the patients followed by a SGLT-2 inhibitor (35%). GLP-1 receptor agonists had a low prescription rate of 2%. Ninety two percent of the patients reported adhering to their glucose lowering prescription.

In relation to diabetes complications among participants with known diabetes at the time of interview, retinopathy and neuropathy was reported in 15% each of the patients while 9% had nephropathy. The proportion of patients with microalbuminuria was 3% and 1% had a history of peripheral artery disease.

## Discussion

The key findings in this international report of screening for and management of glucose perturbations in patients with CAD revealed wide variability between countries, but all showing poor adherence to international guidelines. A striking observation was the low attendance at any form of professional support, such as diabetes educational programs, which may help explain the poor control of all major CVD risk factors among the participants.

Based on self-reported history of diabetes and an OGTT two thirds of patients with CAD had either previously known or newly detected dysglycaemia. To the best of our knowledge this approach to screening for dysglycaemia has not been studied in this systematic way in a coronary population across all six WHO regions. The present result confirms European findings of a poorly practiced screening for dysglycaemia as reported in the EUROASPIRE investigations [10, 11], and a survey by the European Association for Preventive Cardiology, which noted that healthcare professionals despite a high awareness of diabetes as a risk factor provide suboptimal treatment [18].

Compared with the European data in which one third had known T2DM, the prevalence of 44% is higher in this international study. The total proportion of patients with dysglycaemia, (including newly detected IGT and T2DM) is, however, similar at around two thirds illustrating a worldwide failure to systematically investigate and diagnose glucose perturbations in the coronary population. In the present study there was a significant variation in the proportion of patients with known T2DM across regions and countries. Kenya and the UAE reported prevalences of 67% and 69% respectively and the prevalence was also relatively high in the Western Pacific area. This variance is in accordance with the experience of the NCD Risk Factor Collaboration reporting on worldwide trends in the prevalence of diabetes reporting on a highest prevalence in Polynesia and Micronesia, some countries in the Caribbean, Middle East and north Africa, as well as Pakistan and Malaysia [19]. It is, indeed since long, not the least through work of the International Diabetes Federation (IDF) known that the prevalence of T2DM varies considerably across continents and countries [3]. Another explanation for the present findings may be that, in countries with particularly high proportions, such as Kenya, recruitment of patients may have been from centres specializing in diabetes selectively focused on higher risk patients. Whatever the reasons are for the variability between countries it illustrates the compelling need for improved screening for dysglycaemia, not least because the burden of dysglycemia is increasing in low- and middle-income countries [3, 13], and the availability of evidence based cardioprotective glucose lowering drugs that should be prescribed to all patients with CAD and dysglycaemia. According to the NCD Risk Factor Collaboration diabetes treatment has not increased sufficiently or not at all, especially in low- and middle-income countries [19] which is consistent with the results of this international study.

International guidelines recommend that screening for dysglycaemia should be initiated with FPG and HbA1c, performing an OGTT only if any doubt on the glycaemic state remains following these initial tests [8]. In INTERASPIRE the combination of FPG and HbA1c detected 64% of the patients with previously unknown T2DM. The corresponding proportion using HbA1c as the only test was 49% while the majority, 90%, were detected by the combined use of HbA1c and 2hPG. The corresponding proportions in EUROASPIRE V were 70%, 19% and 63% respectively [11]. The discrepancy is explained by a considerably higher diagnostic contribution of HbA1c in the present investigation than in EUROASPIRE. Analytical bias can be ruled out since the analysis of HbA1c was performed in our central laboratory and those countries (China, Indonesia, UAE) that could not transfer blood samples to Helsinki, Finland, their central laboratories were standardized against our central laboratory. A possible reason is that some of the patients are from regions with commonly occurring hemoglobinopathies. Falsely elevated HbA1c may, for example, appear among individuals with HbS β-thalassemia [20]. Higher HbA1c values are also apparent in individuals without diabetes in many non-white populations (Blacks, Asians, and Latinos) [21]. Regardless of the reason it is likely that the use of HbA1c may have resulted in an overdiagnosis of T2DM in INTERASPIRE. In this context the OGTT combining FPG with a 2hPG would be a more reliable diagnostic alternative. The proportion detected with this combination was 74% in INTERASPIRE and 76% in EUROASPIRE V [11]. Further insights in the potential pitfalls with HbA1c in different populations is of course of theoretical interest. A recent analysis of combined data from EUROASPIRE IV and V cohorts does, however, demonstrate that the OGTT is the best option for the combination of diagnostic and prognostic information [22], and is therefore the best available screening tool currently, and the only one that discloses IGT.

Turning to management of diabetes in clinical practice, the general impression of the results is discouraging. This corresponds with reported experiences from Europe [11] and the US [12, 23]. Regarding lifestyle related habits, about two thirds of the present patients were obese or overweight and a similar proportion reported low physical activity. Only a minority had received dietary advice or had been recommended to increase their physical activity. Moreover, of those patients who were smokers at the time of hospitalization only half of them reported that they had abandoned this habit since their recruiting event.

Cardioprotective pharmacological treatment is universally recommended by international guidelines [8, 24–26]. Individual drugs—antiplatelets/anticoagulants, beta-blockers, ACE- or angiotensin receptor inhibitors and lipid lowering drugs—were prescribed, but only 52% of the patients with newly detected dysglycaemia and just 48% of those with known T2DM received all four drug classes, which are all important for mortality reduction [27]. This level of prescribing is lower than in EUROASPIRE V in which all four cardioprotective drug classes were prescribed to 53% of those with newly diagnosed dysglycemia, and 58% of patients with previously known T2DM [11], but considerably better than reports from the US in which 3% and 7% had a combination of all guidelines advocated drugs [12, 23].

The inadequate professional support to help patients achieve a healthy lifestyle and under prescribing of pharmacological treatments, not always up titrated to optimal dosages, is reflected in the poor risk factor control for blood pressure and lipids. This is even more apparent since most of the patients reported that they were compliant with their pharmacological prescriptions even if this may be an overestimation of true compliance. Only about one third of these high risk dysglycaemic CAD patients had blood pressure and LDL-cholesterol within, at the time of the investigation, guideline recommended targets. The most recent guidelines now advocate an even stricter LDL-cholesterol target (< 1.4 mmol/L) [8]. Glucose control in patients with known T2DM was achieved with a variety of traditional glucose lowering agents such as metformin, insulin and sulphonylureas. These treatments were not sufficiently combined and/or dose titrated since the glycaemic control was poor with only half of the patients below the recommended HbA1c level of 7% (53 mmol/mol). The use of cardioprotective glucose lowering drugs, SGLT 2 inhibitors and in particular GLP-1 receptor antagonists was low and is not consistent with recommendations in international guidelines. That SGLT- 2 inhibitors were prescribed to 35% of the T2DM patients should, however, be seen as encouraging. An important reason for the poor risk factor management in INTERASPIRE may be socio-economic or because of ethical-legal issues and that local guidelines in these countries may be inadequate in terms of applicability, clarity, and communication to general practitioners as shown by Owolabi et al. [28]. They observed that less than one in ten persons with diabetes received adequate management and that it improved with higher income of a country. The prescription rate of glucose lowering medications was 51%. In INTERASPIRE glucose lowering medications were prescribed at a higher rate which is potentially an encouraging development in prescriptions for these patients.

Alongside a need for updated national and local guidelines there are other important ways to improve the quality of care. It is recommended that dysglycaemic patients with CAD should attend some sort of educational programme e.g. a nurse-based diabetes school [8]. A minority, 16%, of the patients with diabetes attended such programme with the lowest prevalence in the Philippines (1%) and highest in Poland (36%). In contrast the proportion of glucose self-monitoring was high in all countries (69%). Another factor of potential importance was the professional level of care. Most patients were seen by a cardiologist (88%), who may not see treatment of diabetes as their responsibility, while only one fifth were cared for by an endocrinologist and only 2% were taken care of by a cardiac specialist nurse. Although this relates to local availability, a reasonable outcome of the present findings should be a review of how to deliver multi-disciplinary, evidence-based effective care for this very high-risk group of patients. One effective way would be to follow the European guideline recommendation that reads: “structured education programmes are recommended in patients with diabetes to improve diabetes knowledge, glycaemic control, disease management, and patient empowerment” and “person-centered care is recommended to facilitate shared control and decision-making within the context of person priorities and goals” [8]. These objectives can be reached by establishing nurse-led diabetes schools [29]. This may be particularly important in middle and low-income countries. The PURE study [30] demonstrated that macrovascular complications and cardiovascular mortality are more prevalent in patients in low-income countries than in middle-income countries and lowest in high-income countries. Even if studies from the last two decades reveal a decline in mortality [31–33], studies like PURE and others [13, 30] demonstrate the increasing global burden of diabetes and associated mortality in low- and middle-income countries.

## Strength and weaknesses

A key strength of the INTERASPIRE study is its rigorous data collection, conducted by trained staff using standardized interviews and protocols. These interviews were performed between six months and two years after the index event, allowing sufficient time for guideline-recommended management practices to be implemented. Another strength is the inclusion of countries from all WHO regions, providing standardized data on diabetes and dysglycemia using the OGTT that, to the best of our knowledge, has not been previously reported in an international coronary patient population. Although sample size calculations targeted 400 patients per country to estimate national prevalence with precision, this was not achieved in every country, but for all countries the combined statistical power was excellent for categorizing dysglycaemia and diabetes. The study, planned in 2019 and launched in 2020, faced delays in patient recruitment and a higher non-response rate due to the COVID-19 pandemic and the limitations of hospital-based interviews. Additionally, one country's data had to be excluded from laboratory analyses because local results could not be standardized with our central laboratory. Despite this, the study highlights significant gaps in the screening and management of CAD patients with dysglycemia worldwide.

## Conclusion

The INTERASPIRE study shows that screening for glucose perturbations is completely inadequate, achievement of guideline-recommended lifestyle changes is poor, and pharmacological management is insufficient, all resulting in under-achievement of risk factor treatment targets. HbA1c may be a less accurate screening test in countries with haemoglobinopathies and in certain ethnicities with the risk of over diagnosing diabetes mellitus. In such circumstances the OGTT is the best test. Patients with coronary disease, and especially those with diabetes require professional support to achieve a healthier lifestyle, attainment of risk factor targets and adherence to all cardioprotective medications.

## Supplementary Information

Supplementary Material 1

## Data Availability

Data supporting the findings can be provided by the corresponding author upon a reasonable request.
