# Supplementary material for: Global detection and management of dysglycaemic patients with coronary artery disease results from the INTERASPIRE survey from 14 countries across six WHO regions
Source: Cardiovasc Diabetol. 2025 Aug 11;24:327. doi: 10.1186/s12933-025-02878-3 (PMC12341346; doi:10.1186/s12933-025-02878-3)
Supplement: Supplementary file 1 — Supplementary Material 1 [file 12933_2025_2878_MOESM1_ESM.docx]

**Supplementary Table 1**

Glycaemic states as defined by the World Health Organization (17)

| Diagnostic test | Cut off level according to the World Health Organization | |
| --- | --- | --- |
| Glycated haemoglobin A1c (HbA1c) | DCCT (%) | IFCC (mmol/mol) |
| Diabetes | ≥6.5 | ≥48 |
| Plasma glucose | mmol/L | mg/dL |
| *Normoglycaemia* | | |
| Fasting | 6.1-6.9 | <110 |
| 2-hour postload | <7.8 | <140 |
| *Impaired Fasting Glucose (IFG)* | | |
| Fasting | 6.1-6.9 | 110-125 |
| 2-hour postload | <7.8 | <140 |
| *Impaired glucose tolerance (IGT)* | | |
| Fasting | <7.0 | <126 |
| 2-hour postload | 7.8-11.0 | 140-199 |
| *Diabetes* | | |
| Fasting | ≥7.0 | <126 |
| 2-hour postload | ≥11.1 | 140-199 |
| *Diabetes* | | |
| Fasting | ≥7.0 | ≥126 |
| 2-hour postload | ≥11.1 | ≥200 |
